# HSF1 Activation Mechanisms, Disease Roles, and Small Molecule Therapeutics

**DOI:** 10.7150/ijbs.110447

**Published:** 2025-04-28

**Authors:** Bingwei Zhang, Yumei Fan, Ke Tan

**Affiliations:** 1 Ministry of Education Key Laboratory of Molecular and Cellular Biology; Hebei Research Center of the Basic Discipline of Cell Biology, Hebei Collaborative Innovation Center for Eco-Environment, Hebei Province Key Laboratory of Animal Physiology, Biochemistry and Molecular Biology, College of Life Sciences, Hebei Normal University, Shijiazhuang, 050024, China.; 2 Key Laboratory of Systems Biomedicine (Ministry of Education), Shanghai Center for Systems Biomedicine, Shanghai Jiao Tong University, Shanghai, 200240, China.

**Keywords:** HSF1, inhibitors, activators, cancer, therapeutic strategy

## Abstract

The heat shock factor 1 (HSF1) is a master transcription regulator that orchestrates the expression of heat shock proteins (HSPs) in response to various cellular stresses. Dysfunction of HSF1 contributes to the pathogenesis of a spectrum of acute and chronic diseases, including cancer. Consequently, the modulation of HSF1 activity through the development of small molecules emerges as a promising therapeutic strategy for disease treatment. The activation of HSF1 is a multifaceted process, governed by a complex interplay of regulatory mechanisms, including post-translational modifications, protein-protein interactions, and a balance between its activation and inactivation. Recently, a plethora of compounds, ranging from synthetic to naturally derived, that either inhibit or activate HSF1 was identified, holding considerable potential for the treatment of numerous human diseases. In this comprehensive review, we elucidate the sophisticated mechanisms underlying activation of human HSF1, introduce its role in the etiology of diseases, and provide a comprehensive summary of the inhibitors and activators of HSF1 that have been discovered to date. This review not only offers novel insights for the development of small molecule therapeutics targeting HSF1 but also charts new territories in the design of innovative interventions for the amelioration of disease.

## 1. Introduction

Heat shock response (HSR) is an evolutionarily highly conserved protective process that regulates the expression of molecular chaperones in response to cellular stresses, such as temperature changes, oxidative stresses, lack of glucose, or the accumulation of misfolded proteins 1. The human heat shock factor (HSF) family consists of six identified members: HSF1, HSF2, HSF4, HSF5, HSFX and HSFY 2. Among them, HSF1 is the most ubiquitously expressed HSF, renowned for its swift and robust ability to induce transcription of HSPs 2. These HSPs are essential for cellular recovery from stress-induced damage and are systematically classified into several major families according to their molecular weights, including large HSPs (HSP110 family), HSP90 family (HSPC), HSP70 family (HSPA), chaperonin/HSP60 family (HSPD), HSP40 co-chaperones (DNAJ), and small HSPs (HSPB) 3, 4 . There is an increasing acknowledgment of the therapeutic potential in manipulating HSF1 activity for the development of novel treatments for various diseases. For instance, the activation of HSF1 has demonstrated to exert cytoprotective effects on cardiomyocytes 5, 6, whereas the inhibition of HSF1 could increase the chemosensitivity 7. To realize these therapeutic benefits, the development of inhibitors and activators that specifically target HSF1 is imperative.

In the following sections, we provide an overview of the activation process of human HSF1, discuss its specific roles in human diseases, and categorize the known activators and inhibitors of HSF1. Our aim is to lay the groundwork for the advancement of innovative therapeutic strategies that target HSF1, offering new avenues for clinical intervention.

## 2. The regulatory mechanism of HSF1 activation and inactivation

### 2.1 Glimpse into the process of HSF1 activation and inactivation

**Figure 1:**
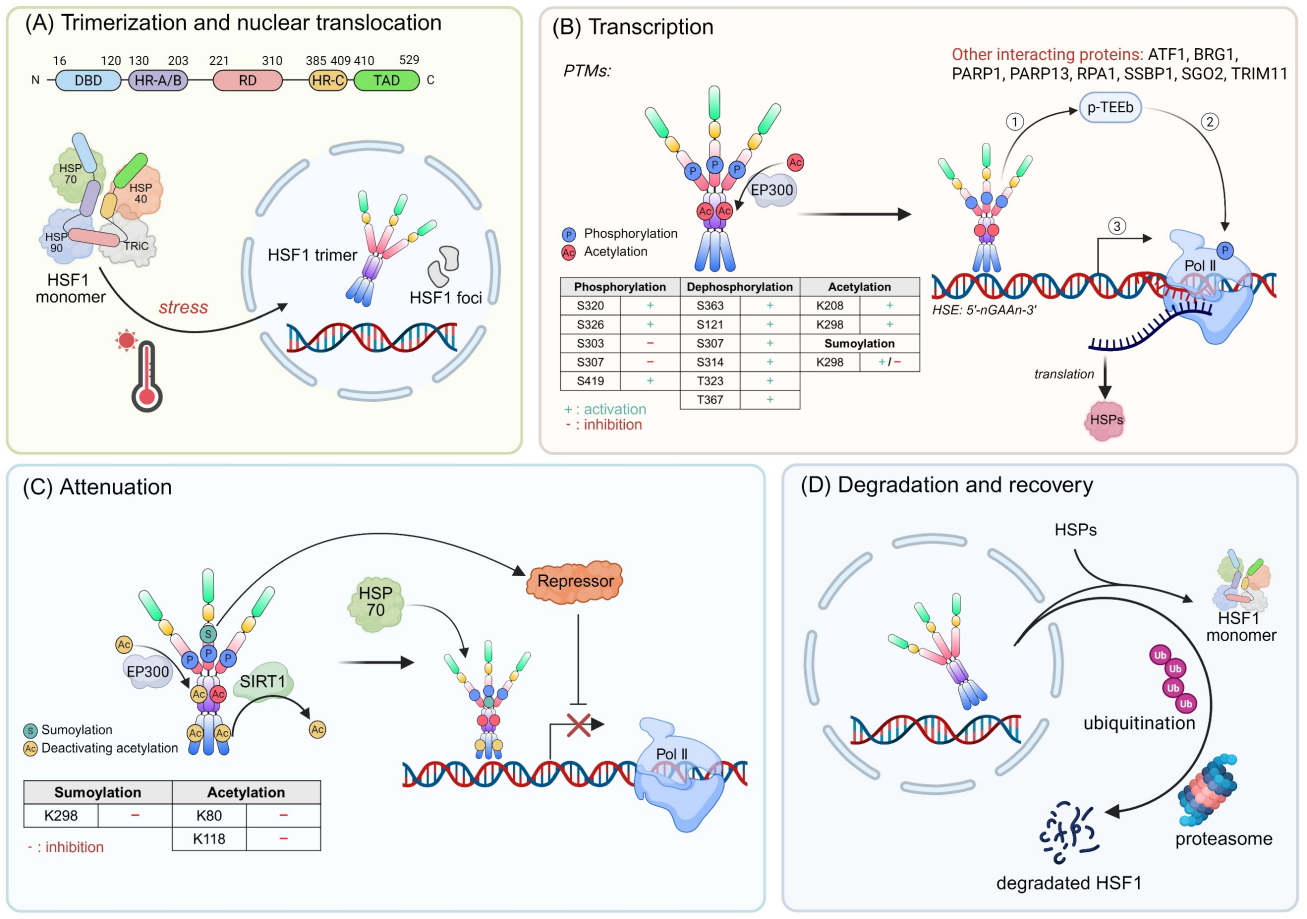
** The activation mechanism of HSF1.** (A) Upper panel: An architectural blueprint of the HSF1 protein. It consists of a DNA-binding domain (DBD), a heptad repeat region (HR) that exhibits leucine-zipper-like characteristics, a regulatory domain (RD), and a trans-activation domain (TAD). Lower panel: Under stress, the quiescent HSF1, which is normally sequestered within a complex with HSPs and the chaperonin TRiC, is released. This liberation triggers a conformational change, leading to the formation of active HSF1 trimers that migrate to the nucleus. (B) The HSF1 trimers subsequently bind to the HSE sequences, thereby initiating the transcription of HSPs. Throughout this transcriptional cascade, the activity of HSF1 is finely tuned by an array of PTMs such as phosphorylation, dephosphorylation, acetylation, and sumoylation, along with interactions from various proteins that can either enhance or repress its activity. (C) The activity of active HSF1 is gradually attenuated through inhibitory acetylation and sumoylation. Additionally, the binding of HSP70 to HSF1 acts as a crucial feedback loop that inhibits HSF1 activity and leads to the cessation of HSP transcription. (D) Following attenuation, HSF1 is released from the DNA and undergoes deactivation. It can either revert to monomeric form, associate with inhibitory protein complexes, or be targeted for ubiquitination and degradation. Figure was created in BioRender.

HSF1 is a multi-domain protein composed of distinct domains that collectively enable its regulatory role. It has an N-terminal DNA-binding domain capable of recognizing and binding to the specific sequences known as heat shock element (HSE) on target gene promoters 8. Additionally, HSF1 contains two heptad repeat regions (HR-A and HR-B), along with a third heptad repeat region (HR-C), which are responsible for its oligomerization 8-11. A regulatory domain is also present, serving as a platform for various post-translational modifications (PTMs) that modulate HSF1 activity 12. Moreover, HSF1 has a C-terminal transactivation domain (TAD) that could recruit the transcriptional cofactors TAF9 and CBP/p300 and initiate the transcription of downstream target genes (Fig. 1A) 13, 14.

Under normal conditions, HSF1 is a cytoplasmic and monomeric molecule that lacks DNA binding activity. Its inactivity is preserved by a cohort of cytosolic HSPs, such as HSP70, HSP90, and chaperonin TCP-1 ring complex (TRiC) (Fig. 1A) 15, 16. Simultaneously, the interactions between HR-A, HR-B and HR-C domains also contribute to the stabilization of HSF1 in its monomeric form 11. Interestingly, even in the absence of stress, the inactive monomeric HSF1 is capable of shuttling between the nucleus and cytoplasm, highlighting the dynamic nature of HSF1 17.

The HSF1 activation during stress responses is a multi-step process that can be delineated into four consecutive phases: (I) Trimerization and Nuclear Translocation: The HR-A and HR-B regions form a highly elongated structure through intermolecular hydrophobic interactions 18. Subsequently, HSF1 undergoes numerous PTMs and interacts with some important regulatory factors, facilitating its conversion to an active trimeric state and nuclear translocation (Fig. 1A) 19. (II) Transcription: Upon translocating into the nucleus, HSF1 acquires DNA-binding activity and binds to the HSEs within promoter regions, thereby inducing the expression of chaperones 8, 20, 21. In yeast genome, transcriptional induction of HSP genes is accompanied by dynamic changes in their 3D structure and spatial organization. For example, Hsf1 can accumulate in distinct nuclear foci (also known as nuclear stress bodies). The formation of these foci is correlated with an enhanced transactivation potential of Hsf1 (Fig. 1B) 22. In another case within the yeast genome, Hsf1, in conjunction with Mediator and RNA polymerase II (Pol II), forms dynamic transcriptional condensates. This process is associated with the 3D reorganization of the genome, which fosters cellular fitness and ensures robust transcriptional activity under stress conditions 23. However, only modest changes in distal regulatory elements on chromatin are observed in human cells following heat shock 24. These results suggests that the global chromatin structure necessary for HSR is preestablished in human enabling cells to respond rapidly to stress. (III) Attenuation: The interaction of HSP70 with HSF1, along with the sumoylation events, contribute to the reduced activity of HSF1 (Fig. 1C) 25-27. (IV) Degradation and Recovery: HSF1 is released from DNA and transformed into monomer with the assistance of the HSP70 system, and this is followed by acetylation and ubiquitination 28. Ultimately, HSF1 is degraded via the ubiquitin-proteasome system (UPS), thus completing the cycle and allowing for cellular recovery (Fig. 1D) 28.

### 2.2 The regulators in HSF1 activation

#### 2.2.1 The PTMs of HSF1

##### 2.2.1.1 Phosphorylation of HSF1

The PTMs of HSF1 include phosphorylation, sumoylation, acetylation and ubiquitination. Notably, phosphorylation events are predominant among these PTMs 29. The PhosphoSitePlus database provides a comprehensive of phosphorylation of HSF1, identifying a total of 59 residues that undergo such modification. Phosphorylation of HSF1 exerts either stimulatory or inhibitory effects on its activation (Fig. 1B) 30, 31. For example, phosphorylation at serine 230 (Ser230) and Ser326 promotes HSF1 activation and are critical for the enhanced interaction between HSF1 and general transcription factors, such as CDK9 and TFIIB 32. In contrast, the constitutive phosphorylation of Ser303 and Ser307 attenuates HSF1 transcriptional activity 33. Moreover, dephosphorylation events can also trigger HSF1 activation. The immediate-early response gene IER5, along with B55 regulatory subunits, induces the dephosphorylation of HSF1 at Ser363, thereby enhancing the expression of HSF1 target genes 34. Additionally, IER5 participates in a ternary complex with phosphatase PP2A and HSF1, dephosphorylating HSF1 at multiple sites, including Ser121, Ser307, Ser314, threonine 323 (Thr323) and Thr367, resulting in the formation of a new hypo-phosphorylated active state of HSF1 35. HSF1 phosphorylation is also related to the active chromatin status and tumorigenesis. A recent study reveals that phosphorylation of HSF1 at Ser419 recruits the TRRAP-TIP60 acetyltransferase complex at the HSP70 promoter during HSR. Subsequently, TRIM33 cooperates with TRIM24 to facilitate mono-ubiquitination of histone H2B at lysine 120 (K120), which stabilizes the HSF1-containing transcription complex in the HSP70 promoter 36.

##### 2.2.1.2 Sumoylation of HSF1

HSF1 undergoes rapid and transient SUMOylation following heat stress 37-39. This dynamic PTM occurs at K298 within a phosphorylation-dependent SUMOylation motif (PDSM), representing the first identified example of this regulatory mechanism 38, 40. SUMOylation efficiency is enhanced by HSF1 trimerization and phosphorylation at Ser303/Ser307, indicating this modification occurs in the transcriptionally active state 25. Non-SUMOylatable mutants (K298R and S303A) of HSF1 exhibit increased transcriptional activity, demonstrating SUMOylation serves as a negative regulatory switch 40. Mechanistically, SUMOylation reduces transactivation potential without affecting DNA binding capacity and HSC70-mediated dissociation from chromatin 25. The PDSM-mediated coupling of phosphorylation and SUMOylation represents a precisely timed attenuation mechanism for HSR. However, another study suggested sumoylation of HSF1 at K298 can enhance its nuclear translocation and stability and HSPs expression, thereby promoting the mitochondrial unfolded protein response (UPR^mt^) activation 41. This process resulted in increased cell proliferation, migration, invasion and reduced apoptosis, ultimately promoting tumor growth in glioblastoma (GBM) xenograft models 41. Nevertheless, a critical limitation of this study is that the K298R mutation concurrently abolishes both SUMOylation and acetylation modifications, making it difficult to definitively attribute the observed effects solely to SUMOylation. Given these conflicting findings, the biological functions of HSF1 SUMOylation need further investigation using more precise genetic tools.

##### 2.2.1.3 Acetylation of HSF1

The acetylation of HSF1 exerts a highly intricate and comprehensive regulatory influence on HSF1 function. The acetylation occurs across a broad spectrum of domains within the HSF1 protein, thereby modulating its activity in a multifaceted manner 9. For instance, under stress conditions, the acetyltransferase EP300 modulates HSF1 stability by acetylating K208 and K298, thereby inhibiting HSF1 degradation and sustaining its activity (Fig. 1B) 28, 42. Conversely, EP300 can also impair the interaction between HSF1 and DNA phosphate backbone by acetylating K80 of HSF1, which attenuated the HSR (Fig. 1C) 28. Similarly, the interplay between HSF1 and BRCA1-associated protein-1 (BAP1) sustains the acetylation status of HSF1 at K80, thereby augmenting the interaction between HSF1 and HSP70 43. This process facilitates the dissociation of HSF1 from chromatin, ultimately suppressing its transcriptional activity 43. However, the NAD-dependent protein deacetylase sirtuin 1 (SIRT1), which deacetylates HSF1 at K80, can counteract this effect by promoting the retention of HSF1 in a DNA-binding state, thereby activating HSPs following heat stress (Fig. 1C) 44. Additionally, acetylation of K118 hinders the binding of HSF1 to chromatin, restricting its DNA binding capacity and initiating the process of attenuation (Fig. 1C) 27, 28.

##### 2.2.1.4 Ubiquitination of HSF1

HSF1 protein is degraded by the UPS during the attenuation phase of HSR (Fig. 1D) 28. The ubiquitin ligase FBXW7 directly binds to HSF1 and mediates its ubiquitination and proteasomal degradation 45. Moreover, the E3 ubiquitin ligase NEDD4 (neuronally expressed downregulated protein 4) promotes HSF1 ubiquitination in neuroblastoma cells overexpressing the A53T mutant variant of α-synuclein 46.

#### 2.2.2 Interactome of HSF1

##### 2.2.2.1 Various types of HSP

HSP70 consistently suppresses HSF1 activity by binding to it and maintaining it in an inactive state (Fig. 1A) 47, 48. Mechanistically, the co-chaperone DNAJB1 aids HSP70 in binding to a region proximal to the HR-B domain of HSF1. This binding induces a conformational flexibility by generating a locally crowded conformation, resulting in a low-entropy state. This process is marked by repeating cycles of entropic pulling, which lead to the dissociation of HSF1 trimers into monomers and the forcibly unzip the triple leucine zipper present within HSF1 trimers 49. Jumonji domain-containing protein 6 (JMJD6) also participates in the regulation of HSF1-HSP70 complex. Specifically, HSF1 binds and promotes the expression of JMJD6, which in turn reduces mono-methylation of HSP70 at arginine 469 (R469), thereby disrupting HSP70-HSF1 repressive complex formation 50. Additionally, the HSP70-HSP40 complex facilitates the interaction between HSP70 and client protein by guiding substrates to the peptide-binding site of HSP70, thereby inducing a conformational change 51, 52. Notably, a recent study suggests that HSP70 also represses Hsf1 by binding to its CE2 domain, a sequence of 14 amino acids, which represses HSF1 cluster formation in yeast 23.

In unstressed cells, HSP90 maintains HSF1 in an inactive state by forming a stable HSP90-HSF1 complex 53, 54. The assembly of HSF1-HSP90 complex suppresses the trimerization of HSF1 (Fig. 1A) 53. HSP90 inhibitors, such as radicicol, celastrol, geldanamycin, and the analogs of geldanamycin (17-AAG and 17-DMAG), target the N-terminal ATP-binding site of HSP90 55-59. This interaction prevents the conformational change of HSP90 to its “closed” dimeric state and competitively disrupts the interaction between HSF1 and HSP90, thus contributing to the transcriptional activation of HSF1 60. Moreover, the HSP90-associated co-chaperone p23 and histone acetyltransferase KAT2A/GCN5 can remove HSF1 and other transcription factors from DNA, although the exact mechanisms remain to be elucidated 27.

TRiC, also referred as CCT (chaperonin containing TCP-1), is composed of multiple subunits. Similar to HSP70 and HSP90, TRiC/CCT interacts with HSF1 and maintains it in an inactive state (Fig. 1A) 16. Specifically, TRiC/CCT is capable of folding substrates within the central cavity of its barrel-like structure, a feature that underpins its chaperone activity 61.

##### 2.2.2.2 Interactions among HSF family members

HSF2 is involved in the developmental processes and spermatogenesis. HSF2 is also implicated in modulating cellular responses to specific proteotoxic stressors, such as proteasome inhibition and febrile-range thermal stress 62, 63. Despite its capacity to bind to consensus HSEs, HSF2 appears to play a relatively limited role in directly promoting the HSR 64. Notably, HSF1 and HSF2 exhibit co-expression across tissues and can form heterotrimers, which functionally impacts gene regulation 64, 65. Recent studies have revealed a physical interaction between HSF2 and HSF1, mediated by their coiled-coil domains 66. This interaction results in similar chromatin occupancy profiles and the coordinated regulation of a shared set of genes. The gene repertoire not only includes HSPs but also encompasses noncanonical transcriptional targets that are crucial for supporting malignant processes 67. Furthermore, a novel interrelationship between HSF1 and HSF2 has been proposed based on findings that HSF1 transcriptionally regulates HSF2 levels following proteasome inhibition 68. This discovery highlights the intricate regulatory network between these two HSFs and their combined roles in cellular stress responses and gene regulation.

HSF1 and HSF4 exhibit distinct and sometimes opposing roles in regulating gene expression and maintaining cellular homeostasis 64, 69. HSF4, particularly its splice variant HSF4b, has been shown to inhibit HSF1 transcriptional activity. By binding to N-terminal hydrophobic region of HSF1, HSF4b disrupts HSF1's intramolecular interactions, promoting its cytoplasmic retention and degradation, thereby attenuating HSF1-driven HSP expression 70. While HSF1 primarily activates HSP genes during stress, HSF4 can suppress this activation by competing for HSE binding or directly inhibiting HSF1's transcriptional function 71, 72. Therefore, HSF1 and HSF4 collaborate or compete in modulating gene expressions, highlighting their context-dependent roles 72, 73. The HSF1-HSF4 relationship exemplifies a sophisticated regulatory mechanism that balances stress adaptation with developmental and homeostatic processes.

##### 2.2.2.3 Transcription factors and other interacting proteins

HSP70 is a classic gene transcribed by Pol II. In the absence of stress, Pol II interacted with DRB sensitivity-inducing factor (DSIF) and negative elongation factor (NELF) to maintain the pausing state 74, 75. However, when subjected to stress, HSF1 recruits transcription factor p-TEFb, which phosphorylates Pol II, DSIF and NELF, thereby removing NELF from Pol II, allowing transcription to proceed 76-78. Thus, the recruitment of p-TEFb contributes to the transcriptional activation of HSF1 (Fig. 1B). In addition, the poly (ADP)-ribose polymerase 1 (PARP1) is liberated from chromatin during heat shock, which is followed by the loss of nucleosomes from the HSP70 coding regions, thereby activating transcriptional process 79-81. Moreover, PARP1 and PARP13 interact with HSF1 to form a ternary complex in response DNA damage, which promotes PARP1 activation and auto-PARylation, and then facilitates the redistribution to genomic lesions 82.

HSF1 recruits diverse transcription factors to modulate gene expression (Fig. 1B). For example, HSF1 recruits mitochondrial single-stranded DNA binding protein 1 (SSBP1) to the promoters of mitochondrial chaperones, subsequently recruiting the chromatin-remodeling factor BRG1. This collaboration is crucial for sustaining mitochondrial membrane potential during proteotoxic stress, thus initiating the UPR^mt^ 83-87. The physical interaction between replication protein A (RPA) and HSF1 is also important. The deletion of RPA1 impairs HSF1's binding to the HSP70 promoter in non-stress conditions, and delays the rapid activation of HSF1 in response to heat shock 88. The nuclear translocation of HSF1 is increased by tripartite motif containing 11 (TRIM11), which interacts with and stabilizes HSF1 89. The pericentromeric adaptor protein shugoshin 2 (SGO2), acting as a coactivator, supports cell survival by promoting the recruitment of Pol II to the HSP70 promoter 90. The phosphorylation of activating transcription factor 1 (ATF1) is essential for the assembly of HSF1 transcription complex during heat shock 91. The ATF1-BRG1 complex facilitates the formation of an active chromatin state and elevates HSP70 expression, and the ATF1-p300/CBP complex accelerates the decline of HSF1 DNA-binding activity during recovery from acute stress, potentially through the acetylation of HSF1 91*.*

## 3. HSF1 is an ideal therapeutic target for human diseases

### 3.1 HSF1 and cancer

**Figure 2:**
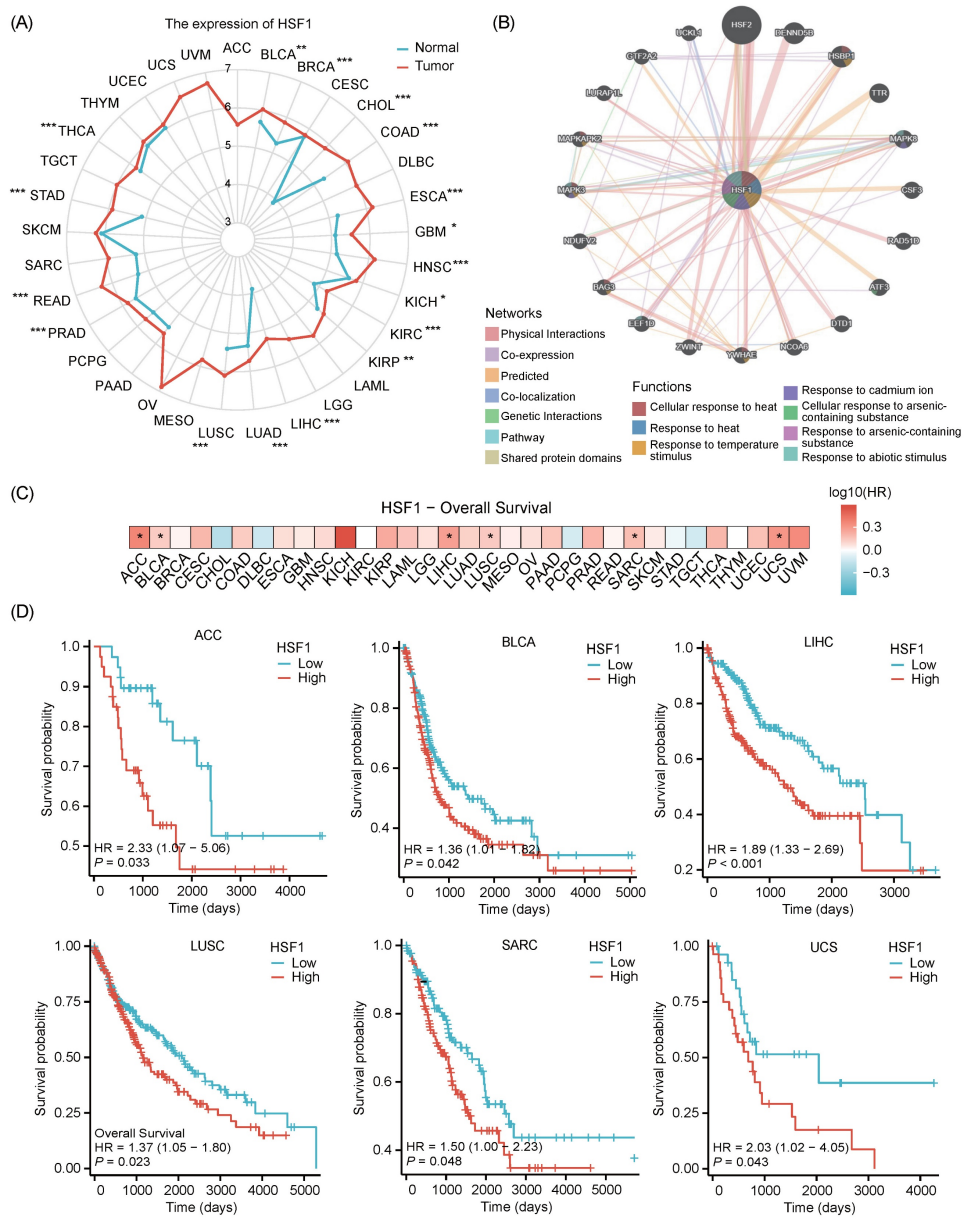
** The expression and prognostic value of HSF1 in pan-cancer.** (A) Radar chart represents the expression of HSF1 in various cancer types. Notably, HSF1 is highly expressed in BLCA, BRCA, CHOL, COAD, ESCA, GBM, HNSC, KICH, KIRC, KIRP, LIHC, LUAD, LUSC, PRAD, READ, STAD and THCA. (B) The HSF1 interactome was generated through GeneMania database analysis, integrating experimental evidence and bioinformatic predictions to produce an unbiased protein-protein interaction (PPI) network. This network offers valuable insights into the interactive partners of HSF1. (C) The prognostic potential of HSF1 in pan-cancer. (D) Kaplan-Meier overall survival (OS) curves are presented to compare the survival rates between patients with high and low HSF1 expression. The elevated HSF1 expression is associated with a poorer prognosis in patients with ACC, BLCA, LIHC, LUSC, SARC, and UCS.

The role of HSF1 in cancer extends far beyond its classical function in stress response. By promoting cancer cell survival, metabolic reprogramming, epithelial-mesenchymal transition (EMT) process and immune evasion, HSF1 emerges as a multifaceted driver of tumorigenesis 92-94. Therefore, targeting HSF1 represents a promising therapeutic strategy. Consistent with previous studies, HSF1 is significantly upregulated in a diverse range of 17 cancer types based on comprehensive analysis of the TCGA database (Fig. 2A) 92, 94. This upregulation is particularly intriguing as HSF1 interacts with a multitude of proteins that are strategically positioned upstream of the tumor growth signaling pathways (Fig. 2B). These interactions suggest a complex regulatory network involving HSF1, which is consistent with its pivotal role in driving the proliferation and growth of cancer cells. The Kaplan-Meier overall survival (OS) curves reveal that patients with low HSF1 expression trend to have longer OS (Fig. 2C, 2D) 95, 96. These findings are consistent with previous pan-cancer analysis, which has implicated a correlation between the elevated HSF1 expression and poor outcomes in patients with different types of cancer 97. These insights reinforce the established connection between HSF1 and tumorigenesis, highlighting its potential as a significant factor in cancer development and progression.

**Figure 3:**
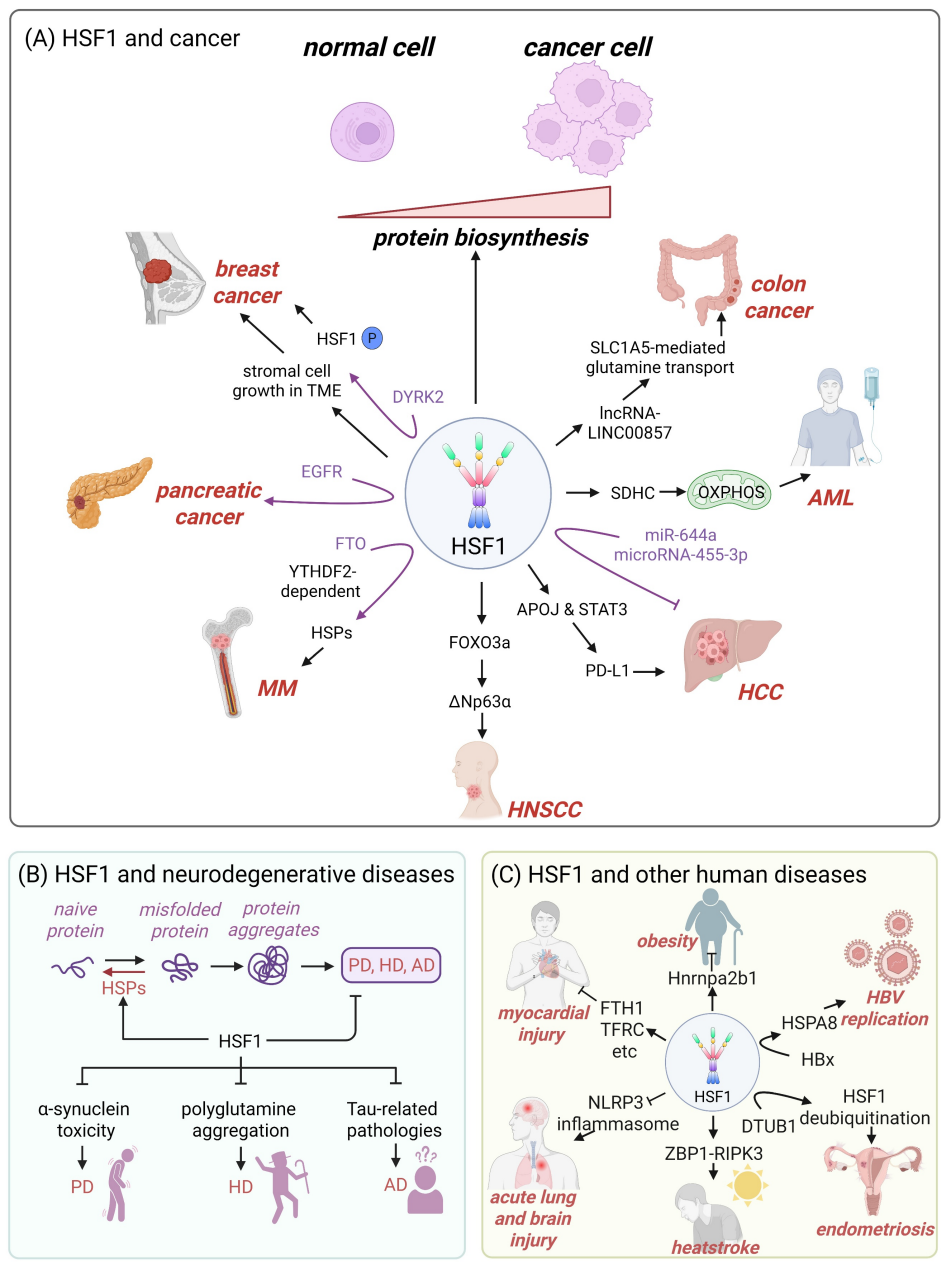
** HSF1 plays a pivotal role in the progression of numerous diseases, highlighting its complex and multifaceted involvement in cellular processes.** (A) In the context of cancer, HSF1 promotes the initiation and progression of the disease by enhancing protein biosynthesis. This increased biosynthetic activity is particularly advantageous for cancer cells, as it supports their rapid growth and proliferation. By upregulating the transcription of genes encoding ribosomal proteins and other factors involved in protein synthesis, HSF1 contributes to the creation of a favorable microenvironment for tumor development. (B) Misfolded protein aggregates lead to the occurrence of neurodegenerative diseases, and the expression of HSPs mediated by HSF1 can exert protective effects by mitigating cellular stress and promoting cell survival, which prevent the progress of this diseases. (C) Beyond cancer and neurodegenerative diseases, HSF1 has also been implicated in a wide range of other human diseases. For instance, it has been shown to play a role in obesity, where it may contribute to metabolic dysregulation and the development of insulin resistance. In myocardial injury, HSF1 activation is linked to the protection of cardiomyocytes from stress-induced damage, suggesting a potential therapeutic target for heart disease. Similarly, in acute lung and brain injury, HSF1-mediated HSPs expression may provide cytoprotective effects, although the precise mechanisms involved remain under investigation. Additionally, HSF1 regulates the expression of genes involved in endometriosis, suggesting a potential role in the disease's pathogenesis. HSF1 is a key player in the progression of multiple diseases, with its effects ranging from promotional to protective depending on the specific context. Figure was created in BioRender.

Cancer cells are tasked with the challenge of increased protein biosynthesis and the buildup of mutated or misfolded proteins 98. HSF1 is capable of enhancing the expression of protein-folding chaperones and an array of pro-survival factors, including DNA replication and repair factors, metabolic enzymes and cell cycle regulators, which increases the malignancy of cancer and shortens the lifespan of cancer patients (Fig. 3A) 21, 94, 99-102. Consistently, previous studies have demonstrated that cancer cells exhibit a tendency to be addicted to HSF1 103, 104.

Accumulating evidence indicates that HSF1 promotes tumor growth across multiple cancer types (Fig. 3A). For instance, in breast cancer, HSF1 stimulates the growth of the stromal cells within the tumor microenvironment (TME), which in turn influences tumor progression 95. DYRK2, a dual specificity tyrosine-regulated kinase, can phosphorylate HSF1, thereby increasing nuclear HSF1 stability and transcriptional potency in breast cancer cells (Fig. 3A) 105. In multiple myeloma, the demethylase fat mass and obesity-associated protein (FTO) exhibits tumor-promoting and pro-metastatic functions by targeting HSF1/HSPs pathway in a m6A reader protein YTHDF2-dependent manner (Fig. 3A) 106. In hepatocellular carcinoma (HCC), HSF1 upregulates PD-L1 expression through inducing apolipoprotein J (APOJ) expression and activating the STAT3 signaling pathway to neutralize the cytotoxic effects of CD8^+^ T cell against cancer cells (Fig. 3A) 107. In acute myeloid leukemia (AML) cells, HSF1 deletion downregulates succinate dehydrogenase C (SDHC) expression and suppresses the mitochondrial oxidative phosphorylation (OXPHOS), thereby inhibiting the growth of tumor cells (Fig. 3A) 108. Additionally, HSF1 also activates the forkhead box protein O3a (FOXO3a)-dependent transcription of ΔNp63α (p63 isoform in the p53 family) to promote the growth of head and neck squamous cell carcinoma (HNSCC) (Fig. 3A) 109. Moreover, epidermal growth factor receptor (EGFR)-HSF1 axis facilitates the initiation of pancreatic cancer (Fig. 3A) 110.

Several microRNAs (miRNAs) regulate the development of human cancers by influencing HSF1 activity. In HCC, miR-644a targets 3'-untranslated region (3'-UTR) of HSF1, leading to a decrease in HSF1 expression, inhibition of HCC cell proliferation and the initiation of apoptosis (Fig. 3A) 111. Similarly, microRNA-455-3p curbs osteosarcoma progression through suppressing HSF1 expression 112. HSF1 also upregulates the acetyltransferase EP300-mediated lncRNA-LINC00857, which facilitates SLC1A5-mediated glutamine transport, ultimately accelerating the growth of colorectal cancer (CRC) cells (Fig. 3A) 113.

### 3.2 HSF1 and neurodegenerative diseases

HSPs are instrumental in preventing the aggregation of misfolded proteins and assisting in the correct folding of newly synthesized polypeptides 114. Therefore, dysfunctional HSF1-mediated downregulation of HSPs expression has been implicated in initiating or exacerbating neurodegenerative diseases, which are characterized by misfolded protein aggregation 19, 115, 116. Targeting HSF1 to increase the expression of HSPs represents a promising therapeutic strategy that may effectively halt or reverse the pathological progression of neurodegenerative disorders while minimizing potential side effects 101, 115. Accumulating evidences suggest that HSF1 mitigates α-synuclein toxicity 46, inhibits the polyglutamine aggregation 117, and prevents Tau-related pathologies 118, thereby delaying the progression of Parkinson's disease (PD), Huntington's disease (HD) and Alzheimer's disease (AD), respectively (Fig. 2B).

PD is characterized by the accumulation of pathological α-synuclein aggregates and microglial overactivation (Fig. 3B) 119, 120. HSF1 has emerged as a critical regulator of neuroprotection in PD, primarily through its induction of HSP70, a chaperone protein that facilitates the clearance of α-synuclein aggregates and modulates microglial activation 121. Furthermore, paeoniflorin, a bioactive monoterpene glycoside, can activate the HSF1-NRF1 (nuclear respiratory factor 1) pathway to mitigate oxidative stress and neuroinflammation in PD 122. These findings highlight HSF1 activation and its downstream effectors as promising therapeutic targets for slowing or preventing PD-associated neurodegeneration.

HD is caused by a CAG repeat expansion in the *HTT* gene, resulting in an elongated polyglutamine (polyQ) tract in the huntingtin protein. This mutation leads to protein misfolding, aggregation, and subsequent neurodegeneration (Fig. 3B) 123. An active form of HSF1 significantly extends the lifespan of HD model mice by suppressing polyQ inclusion formation 117. HSF1 and NFAT (nuclear factor of activated T cells) synergistically promote the expression of the scaffold protein PDZK3 and αB-crystallin, which together contribute to the degradation of polyQ proteins 124. Beyond its role in protein quality control, HSF1 also upregulates brain-derived neurotrophic factor (BDNF) under acute stress, highlighting its neuroprotective function and importance in hippocampal plasticity 125. However, HD pathogenesis involves mechanisms that impair HSF1 activity. Mutant huntingtin upregulates protein kinase CK2α and the E3 ubiquitin ligase FBXW7, both of which promote HSF1 degradation. This loss of HSF1 function exacerbates protein aggregation and striatal neurodegeneration 126. These findings collectively suggest HSF1 activation as a promising therapeutic strategy to counteract HD progression.

AD is neuropathologically defined by extracellular amyloid-β (Aβ) plaques and intracellular tau neurofibrillary tangles (Fig. 3B) 127. In N2a cells stably expressing the pro-aggregation mutant TauRD ΔK280, HSF1 upregulation mitigates tau-induced proteotoxicity through downregulating C/EBP homologous protein (CHOP), a key mediator of ER stress, and enhancing BiP/GRP78 expression to promote proper protein folding 118. Moreover, HSF1 directly counteracts amyloidogenic processes by neutralizing soluble amyloid oligomers through physical interaction 128. HSF1 also protects HSP60 from amyloid oligomer-induced destabilization, thereby preserving mitochondrial proteostasis and preventing mitophagy/apoptosis cascades 128. However, AD progression involves mechanisms that compromise HSF1 function. The non-coding RNA Alu inhibits HSF1-mediated transcription by blocking RNA polymerase II binding at HSF1 target promoters, thereby exacerbating protein misfolding 129. These evidences strongly support the development of HSF1-targeted therapies that could simultaneously mitigate multiple aspects of AD pathogenesis.

### 3.3 HSF1 and other human diseases

Beyond its role in oncology and neurodegenerative disorders, HSF1 is a key player in many other human diseases (Fig. 3C). For obesity treatment, HSF1-mediated upregulation of heterogeneous nuclear ribonucleoprotein A2/B1 (Hnrnpa2b1) results in white fat beige, therefore inducing weight loss by increasing energy expenditure (Fig. 3C) 130. Additionally, overexpression of HSF1 ameliorates palmitic acid-induced myocardial injury by regulating the expression of iron metabolism-related genes, such as FTH1, TFRC, SLC40A1 and GPX4 (Fig. 3C) 5. Notably, HSF1 is also identified as a critical protective factor against various acute and chronic diseases. Recent research has demonstrated that HSF1 protects cells from sepsis-induced acute lung or brain injury by inhibiting the activation of NLRP3 inflammasome (Fig. 3C) 131, 132. Moreover, HSF1 can foster the development of heatstroke by activating the Z-DNA binding protein 1 (ZBP1), which initiates receptor-interacting protein kinase 3 (RIPK3)-dependent cell death (Fig. 3C) 133. In the case of endometriosis, the ubiquitin thioesterase OTUB1 interacts with HSF1, enhancing its protein stability through deubiquitination, and thus contributing to the disease's development (Fig. 3C) 134. Moreover, the hepatitis B X protein (HBx) can upregulate the expression of HSPA8 in an HSF1-dependent manner, which in turn enhances hepatitis B virus (HBV) replication (Fig. 3C) 135.

## 4. HSF1 inhibitors and activators

A vast repertoire of small molecules targeting HSF1, serving as inhibitors or activators, has been identified. These molecules encompass derivatives sourced from natural medicinal compounds, synthetically designed molecules through computational in silico methods, and candidates pinpointed by high-throughput screening of expansive synthetic chemical libraries 136. Notably, the majority of these modulators indirectly regulate HSF1 activity. The intricate mechanisms by which certain molecules act at multiple points within the HSF1 activation cascade have been clearly elucidated, while the precise effects of others remain incompletely understood. Based on the distinct stages at which these molecules operate, we have summarized the inhibitors and activators in Table 1 and Table 2, respectively.

### 4.1 Inhibitors of HSF1

#### 4.1.1 Inhibit HSF1 at the pre-transcriptional level

##### 4.1.1.1 Targeting HSF1-interacting proteins

Doxorubicin (DOX), a widely used anthracycline chemotherapeutic agent originally isolated from *Streptomyces peucetius* 137, exerts its anti-tumor effects through DNA intercalation and Topoisomerase II inhibition. Cardiotoxicity is a major limitation of DOX, primarily due to mitochondrial dysfunction and ROS overload 138. DOX impedes the binding of HSF1 to the carboxyl-terminus of HSP70 interacting protein (CHIP), a co-chaperone and ubiquitin ligase, leading to the destabilization of HSF1 139. Disruption of this interaction by DOX leads to the removal of active HSF1 from the nucleus and triggers its degradation via the proteasome pathway (Table 1) 139. However, the inactivation of HSF1 leads to the loss of its inhibitory effect on insulin-like growth factor receptor II (IGF-IIR), which ultimately accelerates the onset of apoptosis-induced cardiotoxicity 139. These findings not only clarify the molecular mechanisms underlying DOX-induced cardiotoxicity but also reveal that the antitumor efficacy of DOX is partially mediated through suppression of the HSF1 pathway.

##### 4.1.1.2 Inhibitors influencing PTMs of HSF1

Phosphorylation at Ser326 modulates HSF1 activation, and a variety of inhibitors have been designed to target this residue to suppress HSF1 activity. For example, 2,4-bis (4-hydroxybenzyl) phenol, a compound derived from the rhizomes of *Gastrodia elata*, significantly dephosphorylates HSF1 at Ser326, leading to the downregulation of HSP27 and HSP70 expression and the induction of apoptosis in lung cancer cells (Table 1) 140. Bisamide 26 (CCT251236) has inhibitory effects on HSF1-mediated transcriptional activity, leading to a reduction in mRNA expression levels of HSP70 and HSP27. This phenomenon may be associated with its binding affinity to pirin, a redox-sensitive transcription factor regulator. However, the precise implications of this interaction in regulating the HSF1 signaling pathway require further investigation (Table 1) 141. Bisamide 26 exhibits significant antitumor activity by suppressing cancer cell proliferation through the inhibition of the HSF1 pathway 142. It's worth noting that NXP800 (CCT361814), a derivative of Bisamide 26, reduces HSF1 phosphorylation at Ser326, resulting in a concentration-dependent decrease in the expression of HSP27 and HSP70 in myeloma cell lines 143. Importantly, NXP800 has advanced to Phase 1 clinical trials, highlighting its potential as a promising therapeutic agent for refractory ovarian cancer and other malignancies (Table 1) 144.

Additionally, through a comprehensive HSR pathway phenotypic screen of a chemical diversity library with over 100,000 compounds, PW3405, CB14649 and CB01587 are emerged as the most potent HSF1 inhibitors, effectively blocking HSF1 activity by suppressing the phosphorylation at Ser326 (Table 1) 145.

Ser303 and Ser307 are recognized as inhibitory phosphorylation sites on HSF1, and cyclosporin A (CsA) can affect these sites to alleviate HSR 33. CsA, an immunosuppressive agent derived from the fungal species *Tolypocladium inflatum*, exerts its therapeutic effects by binding and inhibiting the activity of calcineurin, thereby disrupting the downstream signaling cascade critical for T cell activation. 146. It enhances the phosphorylation of HSF1 at Ser303 and Ser307 through the ERK1/2, GSK3β and CK2 pathways in heat-treated HeLa cells. Consequently, the transcriptional activity of HSF1 is reduced and the formation of the HSF1-SSBP1 complex is disrupted, leading to downregulated expression of HSPs. This cascade of events ultimately results in a significant decrease in cancer cell survival under hyperthermia or chemotherapy (Table 1) 147. This study establishes a conceptual basis for devising innovative therapeutic approaches for cervical cancer, proposing the integration of CsA in hyperthermic or chemotherapeutic protocols.

Ser320 is an additional target site for HSF1 inhibitors 148. Dorsomorphin, also known as compound C, is a synthesized compound extensively used to inhibit adenosine monophosphate-activated protein kinase (AMPK) 148. Previous studies have indicated that dorsomorphin effectively reduces heat-induced Ser320 phosphorylation and the subsequent nuclear translocation of HSF1 in cancer cells, leading to a reduction of heat-induced HSPs expression in an AMPK-independent manner 149. Moreover, dorsomorphin sensitizes cancer cells to HSP90 inhibitor, downregulates the expression of HSP70, and induces apoptosis of cancer cells, positioning it as a potential therapeutic candidate in oncology (Table 1) 149.

The acetylation and ubiquitination of HSF1 are also strategic targets for inhibitors. For instance, EX527 and AGK2 are synthetic chemical compounds designed to inhibit the deacetylases SIRT1 and SIRT2, respectively 44. Treatment with EX527 or AGK2 decreases the expression of HSF1 and HSP27 through HSF1 deacetylation under both non-stress and heat stress conditions 150. Additionally, these compounds are capable of promoting the ubiquitination of HSF1, which ultimately suppresses cancer cell growth and migration (Table 1) 150. Together, these discoveries suggest that destabilizing the HSF1 protein represents a promising therapeutic approach to inhibit HSF1 activation.

##### 4.1.1.3 Targeting trimerization and nuclear translocation of HSF1

Vitexin, a natural flavone glucoside apigenin found in plants such as *Vitex agnus-castus*, *Phyllostachys nigra*, and *pearl millet*, has been observed to promote nuclear localization of HSF1 while simultaneously impeding HSF1 oligomerization through interaction with its DNA-binding domain (Table 1). Computational modeling indicates that the vitexin-HSF1 complex is stabilized by hydrophobic interactions, which initiates the autophagic cascade and, in turn, suppresses the growth of CRC cell 151. Chelerythrine, a benzophenanthridine alkaloid derived from *Chelidonium majus*, serves as a selective inhibitor of protein kinase C (PKC) 152. Chelerythrine inhibits the nuclear translocation of HSF1 and the expression of HSP70, thereby inducing apoptosis in Dalton's lymphoma cells (Table 1) 153. These results indicate that HSF1 is a viable target for vitexin and chelerythrine in cancer therapy, offering the potential to enhance the effectiveness of chemotherapy.

#### 4.1.2 Inhibiting HSF1 transcriptional activity

##### 4.1.2.1 Targeting DNA binding ability of HSF1

The identification of Direct Targeted HSF1 Inhibitor (DTHIB) through an *in vitro* differential scanning fluorimetry (DSF)-based platform marks a milestone. DTHIB specifically targets the DNA binding domain of HSF1 with a high affinity, selectively accelerating the degradation of nuclear HSF1, while sparing the inactive cytosolic pool. As a result, DTHIB effectively arrested tumor progression in therapy-resistant prostate cancer animal models 154. The selectivity of DTHIB extends to the suppression of HSF1, HSP90 and SDHC, thereby impeding the leukemia stem cell self-renewal in an AML animal model (Table 1) 155. These findings reveal that pharmacological targeting HSF1 holds promise for a broad-spectrum anti-leukemic effect.

The RNA aptamer iaRNA^HSF1^, designed in 2011, binds to HSF1 with an affinity of dissociation constant (Kd) ∼ 8 nM, impeding the binding of HSF1 to HSE (Table 1). In *Drosophila*, iaRNA^HSF1^ induced a reduction in HSP83 level in a HSF1-dependent manner, leading to developmental abnormalities 156. This exemplifies the promise of RNA aptamer technology as a cutting-edge chemical genetic approach for designing small molecules to investigate biological mechanisms.

Several natural compounds possess the capacity to regulate the transcription process of HSF1. Schizandrin A (Sch A), derived from the Chinese medicinal plant *Schisandra chinensis*, exhibits a moderate affinity towards HSF1 through hydrogen bonding, which effectively suppresses HSF1 transcription and the expression of HSPs, thereby inducing apoptosis in CRC cells (Table 1) 157. Quercetin, a plant pigment with potent antioxidant properties, inhibits the heat-induced HSP70 expression by preventing HSF1 from binding to the HSE 158. Additionally, when quercetin is combined with tumor radiofrequency ablation (RFA), a synergistic effect on cancer cell death is observed (Table 1) 159. Fisetin, a natural flavonoid known for its antioxidant and anti-inflammatory properties, blocks the binding of HSF1 to the HSP70 promoter (Table 1) 160. This leads to the suppression of HSP70/BAG3 complex formation and a downregulation of HSP70 expression, culminating in the induction of apoptosis in cancer cells 161.

##### 4.1.2.2 Targeting the recruitment of transcription factors and transactivation function of HSF1

A structure-activity analysis coupled with computational screening led to the discovery of I_HSF_115, a novel HSF1 inhibitor that suppresses HSF1 transcriptional activity by interfering with the assembly of ATF1-containing transcription complexes (Table 1) 162. Moreover, KRIBB11 was discovered by using a synthetic chemical library screening in 2011. Specifically, KRIBB11 inhibits HSF1-dependent recruitment of p-TEFb to the HSP70 promoter to suppress the process of transcription activation and induce apoptosis in cancer cells (Table 1) 163. Notably, KRIBB11 treatment significantly suppresses the lymphatic metastasis in bladder cancer without apparent toxicity 164. Both of SNS-032 and 4,6-pyrimidine series are inhibitors of CDK9 165-167. Because CDK9 is a component of the p-TEFb, SNS-032 and 4,6-pyrimidine series may affect the HSF1-regulated transcription 168, 169. Consistently, recent studies have demonstrated that both compounds inhibit HSF1 transcriptional activity and effectively suppress HSP70 expression in U2OS cells 170, 171. Additionally, 4,6-pyrimidine 25 effectively blocks the expression of HSP70, representing a promising therapeutic strategy for cancer treatment (Table 1) 167. Cantharidin, a compound derived from traditional Chinese medicine 172, not only restrains the binding of HSF1 to the HSP70 promoter, but also hinders the recruitment of HSF1-dependent p-TEFb and blocks the p-TEFb-dependent phosphorylation of C-terminal domain (CTD) of Pol II, thereby arresting the transcriptional elongation and inducing apoptotic cell death in CRC cells (Table 1) 173.

Triptolide, derived from the traditional Chinese herb *Tripterygium wilfordii*, selectively interfered with the proper activity of the C-terminal transactivation domain of HSF1 without affecting its trimerization, hyperphosphorylation, or DNA-binding capacity (Table 1). Additionally, triptolide reversibly inhibits HSP70 mRNA expression and enhances stress-induced cell death in Hela cells 174.

#### 4.1.3 HSF1 inhibitors with unclear mechanisms

To date, the intricate mechanisms underlying the functional exertion of certain inhibitors remain incompletely understood. Our current understanding is limited to the observation that these inhibitors cause a reduction in the expression levels of HSF1 and/or HSPs. While this observation provides some insights into the potential mode of action of these inhibitors, it falls short of offering a comprehensive explanation of their detailed mechanisms. Further researches are necessary to elucidate the full range of interactions and pathways through which these inhibitors exert their effects, potentially involving additional molecular targets and regulatory networks. Such insights could lead to the development of more effective and targeted therapeutic strategies.

##### 4.1.3.1 Natural HSF1 Inhibitors

Sulphoraphane, a naturally isothiocyanate prevalent in cruciferous vegetables, suppresses the expression of HSF1 and its target genes HSP70 and HSP90, ultimately induces apoptosis in breast cancer cells (Table 1) 175. CL-43, a member of the cardenolide family, effectively inhibits the expression of HSP70, HSP90 and HSP40 in HCT-116 cells, potentially through HSF1-mediated transcriptional mechanism. This suppression diminishes the motility and colony formation ability of HCT-116 cells, without exhibiting toxicity towards several normal cell lines (Table 1) 176. Stresgenin B, isolated from a culture supernatant of *Streptomyces spp.*, causes the decreased expression of HSPs, including HSP70, HSP90 and HSP110, and confers moderate cytotoxic effects on several cancer cell lines (Table 1) 177. Ginsenoside Rg3, a steroidal saponin isolated from *Panax ginseng*, downregulates the expression of HSF1 and its downstream target gene fucosyltransferase IV (FUT4) (Table 1) 178. As FUT4 is an essential enzyme in glycolipids fucosylation that promotes cancer cells growth, Rg3 treatment ultimately induces the death of gastric cancer cells 178. Rocaglamide A (Roc A), a natural product isolated from *Aglaia genus*, potently inhibits HSE reporter system with an IC50 of approximately 50 nM (Table 1) 179. Rohinitib (RHT), an analog of Roc A, exhibits greater potency than Roc A in inhibiting the HSE reporter, with an IC50 of around 20 nM 179. RHT abolishes HSF1 binding throughout the genome, resulting in decreased mRNA levels of HSP40 and HSP70 without affecting HSF1 protein levels, ultimately leading to the suppression of tumor growth (Table 1) 179. Given that Roc A also suppresses the activity of the translation initiation factor eIF4A 179, 180, it is suggested that rocaglates including Roc A and RHT may hinder HSF1 function by inhibiting translational flux, including protein synthesis and energy metabolism 179, 180. Homoharringtonine, a natural plant alkaloid derived from *Cephalotoxus fortune*, is a protein synthesis inhibitor and has emerged as a critical therapeutic agent in hematologic malignancies 181, 182. Homoharringtonine directly binds to HSF1, and suppresses its expression and transcription of HSF1 target genes, leading to the selective inhibition of pancreatic cancer cell viability with high HSF1 expression (Table 1) 183.

##### 4.1.3.2 Synthetic HSF1 Inhibitors

KNK437, a benzylidene lactam compound, is recognized as a potent experimental inhibitor of HSF1. KNK437 effectively inhibits the expression of heat-induced HSPs in human CRC cells, including a range of proteins such as HSP27, HSP40, HSP70 and HSP105 (Table 1) 184, 185. VM1, a triazole nucleoside analogue, downregulates the expression of HSF1, HSPs (HSP27, HSP70 and HSP90α) and androgen receptor (AR), offering potential benefits for prostate cancer therapy (Table 1) 186. NZ28, a dehydroemetine derivative, was identified as a potent and non-toxic inhibitor of the HSR through high-throughput chemical screening 170. NZ28 effectively suppresses the induction of HSPs, including HSP70 and HSP27, in various cell lines under various stress conditions (Table 1) 170. Emunin (NZ71), a glycine amide conjugate of emetine, also displays non-toxicity while reduces the expression of HSP70 and HSP27, and significantly increases the sensitivity of myeloma and prostate carcinoma cells to inhibitors of proteasome and HSP90 (Table 1) 170.

### 4.2 Activators of HSF1

#### 4.2.1 Activate HSF1 at the pre-transcriptional level

##### 4.2.1.1 Targeting HSF1-interacting proteins

HSF1A, a potent small-molecule activator of HSF1, mitigates the suppressive effects of the HSF1-TRiC interaction though its direct binding to the TRiC/CCT complex (Table 2). This intervention enhances the expression of HSPs, thereby providing cellular defense mechanisms against protein misfolding and stress-induced apoptosis 16, 115. Additionally, the synergistic application of HSF1A alongside 17-AAG has demonstrated the ability to augment the reconstituting activity of ex-vivo-cultured hematopoietic stem cells (HSCs), ensuring proteostasis and bolstering the regenerative capacity of HSCs resilience during periods of culture-induced stress and senescence 187. Geldanamycin, noted for its specific affinity to HSP90 188, effectively dismantles the stable complex of HSP90/HSF1, thereby activating HSF1 in mammalian cells (Table 2) 53, 189. This activation is linked to neuroprotective effects, offering therapeutic promise in the treatment of HD 53, 189. Phenethyl isothiocyanate (PEITC), abundant in watercress (*Nasturtium officinale*), triggers the dissociation of HSF1 from HSP90 and further induces HSF1 phosphorylation at Ser326 (Table 2) 190. This dual action drives the nuclear accumulation of HSF1 and triggers a marked induction of HSP70, highlighting its potential as a therapeutic agent 190.

##### 4.2.1.2 Targeting PTMs of HSF1

SYSU-3d, a 2-pyrimidinylindole derivative, facilitates the phosphorylation of HSF1 at Ser326 and its nuclear translocation in HCC cells (Table 2) 191. Subsequently, SYSU-3d activates the HSF1/PPARγ coactivator-1α (PGC-1α) pathway, increases mitochondrial biogenesis, combats oxidative stress, and alleviates non-alcoholic steatohepatitis (NASH) 191. Englerin A (EA), derived from the stem bark extract of *Phyllanthus engleri* 192, stimulates the protein kinase C-θ (PKCθ)-dependent phosphorylation of HSF1 at Ser333, leading to the dissociation of HSF1 from HSP90, and enhancing the transcriptional activity of HSF1 (Table 2). EA induces tumor cell glucose dependency and triggers glycolytic cell death under glucose deprivation conditions 193. Resveratrol, a natural phenol synthesized by various plants in response to stress 194, promotes SIRT1-mediated deacetylation of HSF1 and upregulates the expression of HSP25 and HSP70 (Table 2) 195. These actions protect motor neurons from mutant superoxide dismutase 1 (SOD1)-induced neurotoxicity, significantly extend the lifespan of mutant SOD1-bearing mice. These findings suggest that resveratrol may be a prospective therapeutic for amyotrophic lateral sclerosis (ALS) 195.

##### 4.2.1.3 Targeting trimerization and nuclear translocation of HSF1

Celastrol, a chemical compound derived from the root extracts of *Tripterygium wilfordii* and *Tripterygium regelii*, regulates energy metabolism through activating HSF1-PGC1α transcriptional axis 196. Additionally, celastrol is frequently combined with other therapeutic drugs to achieve synergistic antitumor efficacy. For instance, the combination of nanoparticle-encapsulated celastrol with DOX can enhance drug accumulation in multidrug-resistant cells. This is achieved by inducing HSF1 trimerization and nuclear translocation, which in turn promotes autophagy and apoptosis in DOX-resistant cells, pointing to a promising strategy for overcoming DOX resistance via synergistic chemotherapy 197. Similarly, our recent study also reveals that the combination of celastrol with erastin, well-known ferroptosis inducer, synergistically triggers cell death of lung cancer cells via the ROS-mitochondiral fission-mitophagy pathway. Interestingly, the co-treatment of celastrol and erastin also significantly boosts the expression of HSPs by promoting the phosphorylation and nuclear translocation of HSF1. Knockdown of HSF1 further enhances the cytotoxic of combination of erastin and celastrol *in vitro* and *in vivo* 198. Collectively, these observations clearly highlight the role of celastrol as a potential activator of HSF1, with significant implications for cancer therapy (Table 2).

Curcumin, a natural product extracted from the rhizome of the turmeric plant (*Curcuma longa*) 199, induces HSF1 trimerization 200, promotes its nuclear translocation 201, and enhances the DNA binding capacity, leading to increased HSP70 expression in human colorectal carcinoma 200 and HeLa cells (Table 2) 201 . Additionally, curcumin specifically accelerates HSP90 mRNA degradation in *C. albicans* 202, which may contribute to the activation of HSF1. Oridonin, a natural terpenoids derived from the traditional Chinese medicinal herb *Isodon rubescens*, triggers oxidative stress in cancer cells by directly binding to the C153 residue of HSF1 203. This interaction prevents the formation of intramolecular disulfide covalent bonds and instead promotes the formation of intermolecular disulfide covalent bond formation, facilitating HSF1 trimerization and increasing the expression of HSP70 and ubiquitin proteins (Table 2) 203. Metformin, a biguanide that is extracted from the herb *Galega officinalis*, is the first-line therapy for type 2 diabetes mellitus due to its efficacy, safety profile, and cost-effectiveness 204. Metformin promotes the nuclear translocation of HSF1 and activation of HSF1-UPR^mt^ signaling pathway, providing a cardioprotective effect under pressure overload conditions 205. Calycosin is an O-methylated isoflavone isolated from *Astragalus membranaceus* Bge 206. A recent study indicates that HSF1 can translocate to the nucleus to enhance cell survival following calycosin treatment (Table 2) 207.

#### 4.2.2 Activate HSF1 at the transcriptional level

Bimoclomol, a non-toxic hydroxylamine derivative with therapeutic potential in diabetes and cardiac dysfunction, exhibits a subtle yet significant binding affinity to HSF1. This interaction prolongs HSF1's residence time on the HSE and triggers a moderate phosphorylation of HSF1 in response to cellular stress, thereby activating the HSR (Table 2) 208-210. FLZ, synthesized from the Chinese herb *Annona glabra*, facilitates the nuclear translocation of HSF1 and promotes its binding to the HSEs (Table 2) 211. Importantly, FLZ treatment significantly upregulates the expression of HSP27 and HSP70, conferring a robust neuroprotective effect in experimental models of PD 211.

#### 4.2.3 Activate HSF1 at post-translational level

Proteasome inhibitors, such as MG132 and bortezomib, are used as clinical drugs for treatment of myeloma. These drugs stabilize HSF1 by inhibiting its proteasomal degradation (Table 2) 212. Moreover, these agents promote the differentiation of CD69^+^ regulatory T cells (Tregs), resulting in the efficient production of Tregs for the treatment of colitis and other autoimmune diseases characterized by Tregs deficiency 212.

#### 4.2.4 The activators with unclear mechanisms

Tanshinone IIA (TIIA) is a compound derived from the traditional medicinal herb *Salvia miltiorrhiza* with prominent anticancer properties. TIIA induces the expression of HSF1 and phosphorylation of HSP27 at Ser82 in gastric cancer cells (Table 2) 213. This triggers a cascade that includes the accumulation of ROS, the activation of UPR, and the cell death in gastric cancer 213. Paeoniflorin, derived from herb *Paeonia lactiflora*, increases the expression of HSF1 and HSP70, suppresses protein aggregation in neuronal differentiated SH-SY5Y cells, and improves the neurodegenerative symptoms in animal models of PD and AD (Table 2) 214. Total ginsenoside (TG), a mixture of the primary active ginsenosides from *Panax ginseng*, upregulates the expression of HSF1 and HSP-16.2 in *C. elegans*. One of its main constituents, ginsenoside Rd, extends the lifespan of *C. elegans*, indicating the involvement of TG in HSF1-mediated aging (Table 2) 215. U-133, an acetylated tris-O-glucoside echinochrome and the main pigment in sea urchins, activates HSF1 to an extent akin to mild heat shock, promoting HSP70 expression (Table 2). This activation counters PD-like neurodegeneration 216. Taurine, an amino sulfonic acid abundant in fish, upregulates the myocardial expression of HSF1 and HSP70, thereby exerting cardioprotective effects (Table 2) 6. TRC051384, a compound from the substituted 2-propen-1-one class, is a potent inducer of HSP70. It significantly attenuates stroke-associated neuronal impairment and dysfunction in a rat model of transient ischemic stroke (Table 2) 217. Astragaloside IV (AS-IV), a natural product isolated from *Astragalus membranaceus* Bge, elevates the expression of HSF1 at both the mRNA and protein levels (Table 2) 218. Additionally, AS-IV increases the expression levels of HSP70, HIF-1α and VEGF, collectively contributing to an amelioration of heart failure induced by pressure overload 218.

## 5. Discussion

HSF1 stands as a pivotal transcriptional regulator that orchestrates the induction of HSPs, serving as a protective mechanism for proteomic quality control (PQC) and maintenance of protein homeostasis in response to stress 219, 220. The successful isolation, cloning and identification of HSF1 have marked significant milestones, propelling research in this domain to rapidly progress over the past three decades 221. The elaborate assembly of multi-protein complexes and HSF1-mediated transcriptional activation are cornerstones of cellular PQC mechanisms 221, 222. Disruptions or aberrant activation of HSF1 pathways can precipitate a cascade of events that destroy the physiological and pathological balance, leading to dysfunction or abnormality in protein PQC 101, 221. Such imbalances are implicated in the etiology of cancer 98, neurodegenerative diseases 19, 115 and a spectrum of other human diseases 5, 130, 131, 133, 134.

The discovery of HSF1 has not only deepened our understanding of the cellular stress response but has also unveiled potential therapeutic targets for various diseases. The activation of HSF1 and the subsequent upregulation of HSPs can protect cells from stress-induced damage, offering a preventive or mitigating effect against the progression of certain pathologies 19, 115. Conversely, the dysregulation of HSF1 activity, such as sustained overactivation or insufficient activation, contributes to pathological outcomes 19, 115. For instance, in neurodegenerative diseases, the failure to mount an adequate stress response leads to the accumulation of misfolded proteins and cellular toxicity 19, 115. Moreover, the intricate interplay between HSF1 and other cellular pathways adds layers of complexity to its role in disease 95, 100, 223. The modulation of HSF1 activity, therefore, needs to be finely balanced to harness its potential therapeutic benefits without exacerbating pathological conditions. Ongoing research is focused on elucidating these complex mechanisms to develop targeted therapies that can modulate HSF1 activity in a controlled manner.

In the current landscape of pharmacological research, a multitude of inhibitors and activators that target different stages of HSF1 activation have emerged, demonstrating significant therapeutic potential in experimental settings. As research progresses, the development of HSF1 modulators will likely benefit from advances in structural biology, computational modeling, and high-throughput screening technologies. These tools facilitate the design of molecules with improved binding affinity, selectivity, and pharmacokinetic properties. Despite their promise, the majority of small molecules face the challenge of lacking specificity and potency due to their inability to bind directly to HSF1. However, a select group of HSF1 inhibitors has been identified that can interact directly with HSF1, including vitexin 151, DTHIB 154, Sch A 157, I_HSF_115 162, bimoclomol 208, oridonin 42 and HHT 181. These selective inhibitors have opened new avenues in the development of HSF1 modulators, offering more precise control over HSF1 activity. Additionally, the RNA aptamer technology, exemplified by iaRNA^HSF1^, represents a promising chemical genetic approach to discovering novel HSF1 inhibitors. This technology harnesses the specificity of nucleic acid hybridization to modulate protein function, providing a new frontier in the search for HSF1 inhibitors. In addition to direct HSF1 modulators, many inhibitors of HSP70 and HSP90, such as Timosaponin AIII (Tim-AIII) 224, C0818 225, and VER-155008 226-228, have been identified. While these compounds were initially discovered for their effects on HSPs, their potential to modulate HSF1 activity warrants further exploration, as they may indirectly influence the HSR by affecting the expression or function of HSPs. The exploration of these diverse compounds necessitates a comprehensive understanding of the complex interplay between HSF1 and other cellular pathways. It is essential to elucidate how these inhibitors and activators integrate into the intricate network of cellular responses to stress and how they might be leveraged to achieve therapeutic outcomes. Moreover, the translation of these findings from the laboratory to the clinic will require rigorous preclinical and clinical evaluation to assess the safety, efficacy and optimal dosing regimens of these compounds. The goal is to develop therapies that can be tailored to individual patient needs, taking into account factors such as disease stage, genetic background and comorbidities.

In summary, the pivotal role of HSF1 in upholding cellular proteostasis and its profound implications in a diverse array of diseases highlight the imperative for ongoing research. The quest for the discovery and refinement of HSF1 modulators stands as a dynamic and promising field, with the capacity to revolutionize therapeutic approaches for diseases characterized by protein misfolding and aggregation. The advancement of HSF1-targeted therapies is expected to benefit from interdisciplinary collaboration, merging insights from molecular biology, pharmacology, and genomics. The integration of bioinformatics and systems biology will further enhance our ability to predict the effects of HSF1 modulation and to identify potential off-target effects, thereby facilitating the design of safer and more effective drugs.

## Figures and Tables

**Table 1:** Summary of HSF1 inhibitors

Compounds	Chemical structure	Source	Mechanism of action
*Targeting DNA binding ability of HSF1*			
Doxorubicin (DOX)	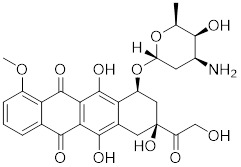	*Streptomyces peucetius*	Impedes the binding of HSF1 to CHIP, leading to the destabilization and degradation of HSF1 139.
2,4-bis(4-hydroxybenzyl) phenol	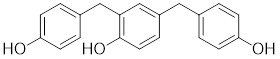	*Gastrodiaelata*	Dephosphorylates HSF1 at Ser326 and decreased the expression of HSP27 and HSP70 140.
NXP800 (CCT361814)	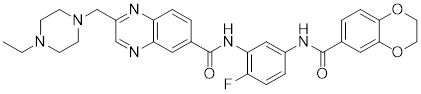	Derivatives of bisamide (CCT251236)	Suppresses HSF1 phosphorylation at Ser326 and decreased the expression of HSP27 and HSP70 143.
PW3405	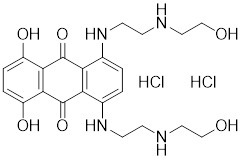	A compound identified by phenotypic screen	Suppresses the phosphorylation of HSF1 at Ser326 145.
CB14649	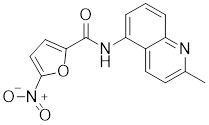	A compound identified by phenotypic screen	Suppresses the phosphorylation of HSF1 at Ser326 145.
CB01587	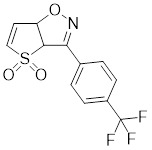	A compound identified by phenotypic screen	Suppresses the phosphorylation of HSF1 at Ser326 145.
Cyclosporin A (CsA)	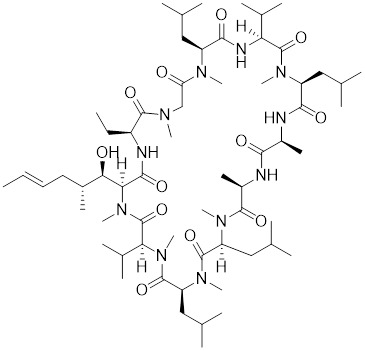	*Tolypocladiuminflatum*	Enhances the phosphorylation of HSF1 at Ser303 and Ser307 147.
Dorsomorphin	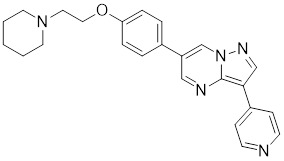	A synthesized compound that inhibits AMPK	Reduces heat-induced Ser320 phosphorylation and nuclear translocation of HSF1 149.
EX527	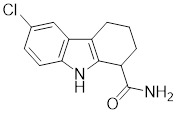	A SIRT1 inhibitor	Promotes HSF1 deacetylation and ubiquitination 44.
AGK2	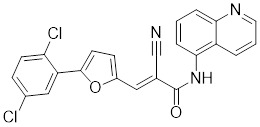	A SIRT2 inhibitor	Promotes HSF1 deacetylation and ubiquitination 44.
*Targeting trimerization and nuclear translocation of HSF1*			
Vitexin	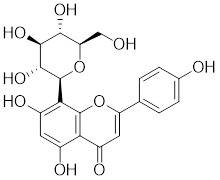	*Vitex agnus-castus*	Impedes HSF1 oligomerization by interacting with its DNA-binding domain 151.
Chelerythrine	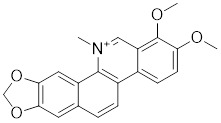	*Chelidonium maju*	Suppresses the nucleus translocation of HSF1 and expression of HSP70 153.
DTHIB	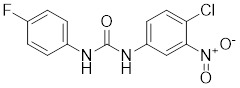	*In vitro* screen targeting recombinant human HSF1 DBD	Specifically targets the DBD of HSF1 and accelerates nuclear HSF1 degradation 154.
Schizandrin A (Sch A)	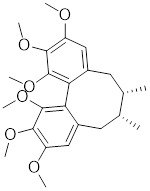	*Schisandra chinensis*	Suppresses HSF1-regulated transcription and expression of HSPs 157.
Quercetin	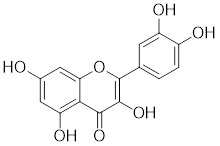	A plant pigment	Prevents HSF1-HSE binding and inhibits heat-induced HSP70 expression 158.
Fisetin	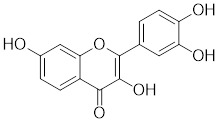	A natural flavonoid	Blocks the binding of HSF1 to the HSP70 promoter 160.
*Targeting HSF1-dependent recruitment of transcription factors*			
I_HSF_115	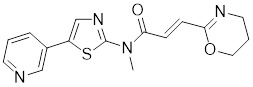	A structure-activity analysis couple with computational screening	Interferes with ATF1-containing complex assembly, and dampens HSF1 activity 162.
KRIBB11	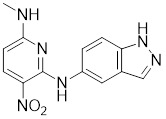	A synthetic chemical library screening	Inhibits HSF1-dependent recruitment of p-TEFb to the HSP70 promoter 163.
SNS-032	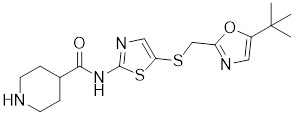	Inhibitor of CDK9	Inhibits HSF1 transcriptional activity and HSP70 expression 170, 171.
4,6-pyrimidine 25	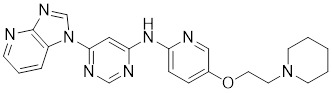	Inhibitor of CDK9	Inhibits HSF1 transcriptional activity and HSP70 expression 167.
Cantharidin	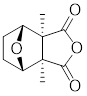	Secreted by many species of blister beetles	Blocks HSF1 binding to HSP70 promoter and prevents p-TEFb recruitment 173.
*Targeting the transactivation function of HSF1*			
Triptolide	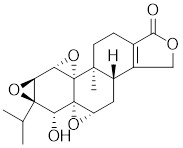	*Tripterygium wilfordii*	Interferes with the activity of the C-terminal transactivation domain of HSF1 174.
*HSF1 inhibitors with unclear mechanisms*			
Sulphoraphane	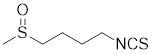	A naturally isothiocyanate	Suppresses the expression of HSF1, HSP70 and HSP90 175.
CL-43	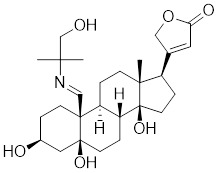	A member of the cardenolide family	Suppresses the expression of HSP70, HSP90 and HSP40 176.
Stresgenin B	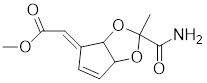	*Streptomyces sp*. AS-9	Suppresses the expression of HSPs 177.
Ginsenoside Rg3	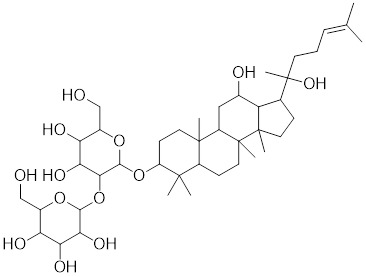	*Panax ginseng*	Suppresses the expression of HSF1 and FUT4 178.
Rocaglamide A (Roc A)	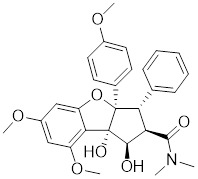	*Aglaia genus*	Inhibits HSE reporter system with an IC50 of approximately 50 nM 179.
Homoharringtonine	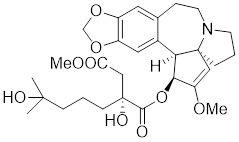	*Cephalotoxus fortunei*	Directly binds to HSF1 and suppresses expression of HSF1 and HSPs 183.
Rohinitib (RHT)	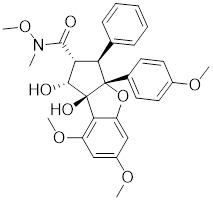	Rocaglamide A analogs	Inhibits HSE reporter system with an IC50 of approximately 20 nM 179.
KNK437	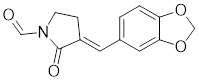	A benzylidene lactam compound	Suppresses the expression of HSPs 184, 185.
VM1	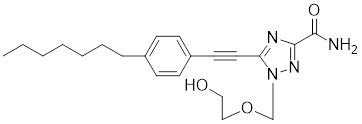	A triazole nucleoside analogue	Suppresses the expression of HSF1 and HSPs 186.
Bisamide 26 (CCT251236)	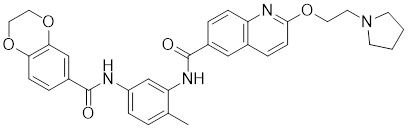	A chemical probe	Suppresses the expression of HSP70 and HSP27 141.
NZ28	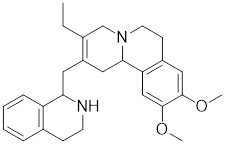	A dehydroemetine derivative	Suppresses the expression of HSP70 and HSP27 170.
Emunin (NZ71)	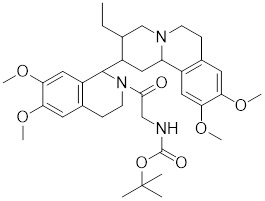	A glycine amide conjugate of emetine	Suppresses the expression of HSP70 and HSP27 170.

**Table 2:** Summary of HSF1 activators

Compounds	Chemical structure	Source	Mechanism of action
*Targeting HSF1-interacting proteins*			
HSF1A	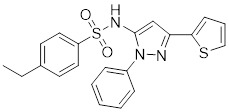	A cell-permeable pyrazolylsulfonamide compound	Disrupts HSF1-TRiC complex through directly binding to TRiC 16, 115.
Geldanamycin	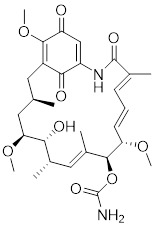	HSP90 inhibitor	Efficiently disrupts the stable HSP90/HSF1 complex by targeting HSP90 53, 189.
Phenethyl isothiocyanate (PEITC)	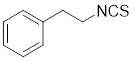	*Nasturtium officinal*	Triggers the dissociation of HSF1 from HSP90 and promotes HSF1 phosphorylation at Ser326 190.
*Targeting PTMs of HSF1*			
SYSU-3d	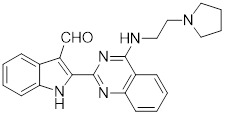	2-pyrimidinylindole derivative	Promotes the phosphorylation of HSF1 at Ser326 and its nuclear translocation 191.
Englerin A (EA)	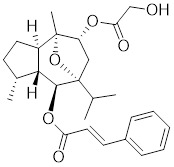	*Phyllanthus engleri*	Triggers PKCθ-mediated phosphorylation of HSF1 at Ser333, causing HSF1 dissociation from HSP90 193.
Resveratrol	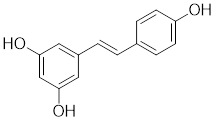	A natural phenol or polyphenol	Promotes SIRT1-mediated deacetylation of HSF1 and upregulates the expression of HSP25 and HSP70 195.
*Targeting trimerization and nuclear translocation of HSF1*			
Celastrol	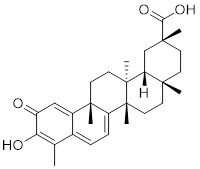	*Tripterygium wilfordii*and *Tripterygium regelii*	Activating HSF1-PGC1αaxis to regulate energy metabolism 196.
Curcumin	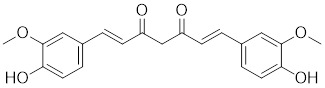	*Curcuma longa*	Enhances HSF1 polymerization, nuclear translocation, and DNA binding ability 200, 201
Oridonin	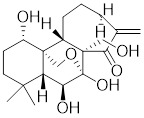	*Isodonrubescens*	Directly binding to the Cys153 of HSF1 and promotes HSF1 trimerization 203.
Metformin	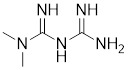	*Galega officinalis*	Promotes HSF1 nuclear translocation and activates HSF1-UPRmt pathway 205.
Calycosin	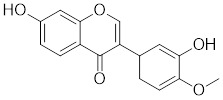	*Astragalus membranaceus* Bge	Promotes HSF1 translocate to nucleus 207.
*Activate HSF1 at the transcriptional level*			
Bimoclomol	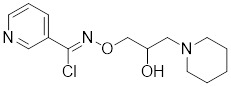	A non-toxic hydroxylamine derivative	Enhances HSF1's binding to HSE and induces moderate HSF1 phosphorylation 208-210.
FLZ	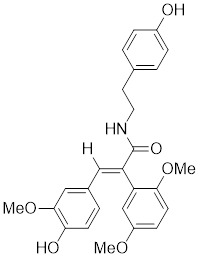	*Annona glabra*	Enhances HSF1 nuclear translocation and DNA binding 211.
*Activate HSF1 at post-translational level*			
MG132	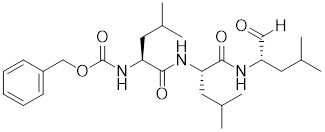	Proteasome inhibitor	Stabilizes HSF1 by inhibiting its proteasomal degradation 212.
bortezomib	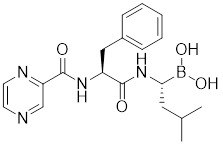	Proteasome inhibitor	Stabilizes HSF1 by inhibiting its proteasomal degradation 212.
*The activators with unclear mechanisms*			
Tanshinone IIA (TIIA)	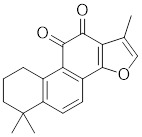	*Salvia miltiorrhiza*	Increases the expression of HSF1 and the phosphorylation of HSP27 at Ser82 213.
Paeoniflorin	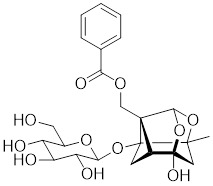	*Paeonia lactiflora*	Increases the expression of HSF1 and HSP70 214.
Ginsenoside Rd	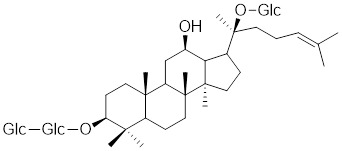	*Panax ginseng.*	Increases the expression of HSF1 and HSP 215.
U-133	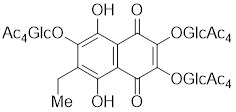	An acetylated tris-O-glucoside echinochrome	Mildly activates HSF1 and enhances HSP70 expression 216.
Taurine	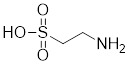	An amino sulfonic acid in fish	Increases the myocardial expression of HSF1 and HSP70 6.
TRC051384	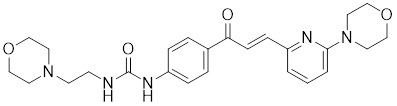	A derivative of 2-propen-1-one	Increased the expression of HSP70 217.
Astragaloside IV	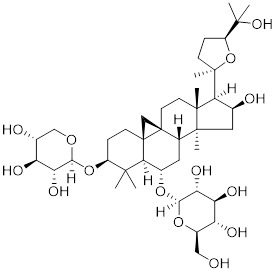	*Astragalus membranaceu* Bge	Upregulates the expression of HSF1 and HSP70 218.

## Abbreviations

3'-UTR: 3'-untranslated region
ACC: Adrenocortical carcinoma
AD: Alzheimer's disease
AMPK: adenosine monophosphate-activated protein kinase
APOJ: apolipoprotein J
AS-IV: Astragaloside IV
ATF1: activating transcription factor 1
Aβ: amyloid-β
BAP1: BRCA1-associated protein-1
BDNF: brain-derived neurotrophic factor
BLCA: Bladder urothelial carcinoma
BRCA: Breast invasive carcinoma
CCT: chaperonin containing TCP-1
CESC: Cervical squamous cell carcinoma and endocervical adenocarcinoma
CHIP: carboxyl-terminus of HSP70 interacting protein
CHOL: Cholangio carcinoma
CHOP: C/EBP homologous protein
COAD: Colon adenocarcinoma
CRC: colorectal cancer
CTD: C-terminal domain
DLBC: Lymphoid neoplasm diffuse large B-cell lymphoma
DOX: Doxorubicin
DSF: differential scanning fluorimetry
DSIF: DRB sensitivity-inducing factor
DTHIB: Direct Targeted HSF1 Inhibitor
EA: Englerin A
EGFR: epidermal growth factor receptor
EMT: epithelial-mesenchymal transition
ESCA: Esophageal carcinoma
FTO: fat mass and obesity-associated protein
FUT4: fucosyltransferase IV
FOXO3a: forkhead box protein O3a
GBM: glioblastoma
GBM: Glioblastoma multiforme
HBx: hepatitis B X protein
HBV: hepatitis B virus
HCC: hepatocellular carcinoma
HD: Huntington's disease
Hnrnpa2b1: heterogeneous nuclear ribonucleoprotein A2/B1
HNSC: Head and neck squamous cell carcinoma
HSCs: hematopoietic stem cells
HSF: human heat shock factor
HSF1: heat shock factor 1
HSE: heat shock element
HSPs: heat shock proteins
HSR: Heat shock response
IGF-IIR: insulin-like growth factor receptor II
JMJD6: Jumonji domain-containing protein 6
Kd: dissociation constant
KICH: Kidney chromophobe
KIRC: Kidney renal clear cell carcinoma
KIRP: Kidney renal papillary cell carcinoma
LAML: Acute myeloid leukemia
LGG: Brain lower grade glioma
LIHC: Liver hepatocellular carcinoma
LUAD: Lung adenocarcinoma
LUSC: Lung squamous cell carcinoma
miRNAs: microRNAs
MESO: Mesothelioma
NEDD4: neuronally expressed downregulated protein 4
NELF: negative elongation factor
NFAT: nuclear factor of activated T cells
NRF1: nuclear respiratory factor 1
OS: overall survival
OV: Ovarian serous cystadenocarcinoma
PAAD: Pancreatic adenocarcinoma
PARP1: poly (ADP)-ribose polymerase 1
PCPG: Pheochromocytoma and paraganglioma
PD: Parkinson's disease
PDSM: phosphorylation-dependent SUMOylation motif
PEITC: Phenethyl isothiocyanate
PGC-1α: PPARγ coactivator-1α
PKC: protein kinase C
Pol II: RNA polymerase II
polyQ: polyglutamine
PQC: proteomic quality control
PRAD: Prostate adenocarcinoma
PTMs: post-translational modifications
READ: Rectum adenocarcinoma
RFA: radiofrequency ablation
RHT: Rohinitib
RIPK3: receptor-interacting protein kinase 3
Roc A: Rocaglamide A
RPA: replication protein A
SARC: Sarcoma
Sch A: Schizandrin A
SDHC: succinate dehydrogenase C
SGO2: shugoshin 2
SIRT1: sirtuin 1
SKCM: Skin cutaneous melanoma
SOD1: superoxide dismutase 1
SSBP1: single-stranded DNA binding protein 1
STAD: Stomach adenocarcinoma
TAD: transactivation domain
TG: Total ginsenoside
TGCT: Testicular germ cell tumors
THCA: Thyroid carcinoma
THYM: Thymoma
TIIA: Tanshinone IIA
Tim-AIII: Timosaponin AIII
TME: tumor microenvironment
TRiC: TCP-1 ring complex
TRIM11: tripartite motif containing 11
Tregs: regulatory T cells
UCEC: Uterine corpus endometrial carcinoma
UCS: Uterine carcinosarcoma
UPS: ubiquitin-proteasome system
UPRmt: mitochondrial unfolded protein response
UVM: Uveal melanoma
ZBP1: Z-DNA binding protein 1
ΔNp63α: p63 isoform in the p53 family
